# Identification of Nrl1 Domains Responsible for Interactions with RNA-Processing Factors and Regulation of Nrl1 Function by Phosphorylation

**DOI:** 10.3390/ijms22137011

**Published:** 2021-06-29

**Authors:** Barbora Mikolaskova, Matus Jurcik, Ingrid Cipakova, Tomas Selicky, Jan Jurcik, Silvia Bagelova Polakova, Erika Stupenova, Andrej Dudas, Barbara Sivakova, Jana Bellova, Peter Barath, Lucia Aronica, Juraj Gregan, Lubos Cipak

**Affiliations:** 1 Cancer Research Institute, Biomedical Research Center, Slovak Academy of Sciences, Dubravska Cesta 9, 845 05 Bratislava, Slovakia; barbora.mikolaskova@savba.sk (B.M.); matus.jurcik@savba.sk (M.J.); ingrid.cipakova@savba.sk (I.C.); tomas.selicky@savba.sk (T.S.); jan.jurcik@savba.sk (J.J.); 2 Institute of Animal Biochemistry and Genetics, Centre of Biosciences, Slovak Academy of Sciences, 840 05 Bratislava, Slovakia; silvia.bagelova@savba.sk (S.B.P.); erika.stupenova@savba.sk (E.S.); 3 Department of Molecular Biology, Faculty of Natural Sciences, Comenius University, Ilkovicova 6, 842 15 Bratislava, Slovakia; dudas@uniba.sk; 4 Institute of Chemistry, Slovak Academy of Sciences, Dubravska Cesta 9, 845 38 Bratislava, Slovakia; chembsiv@savba.sk (B.S.); jana.bellova@savba.sk (J.B.); peter.barath@savba.sk (P.B.); 5 Medirex Group Academy, n.o., Jana Bottu 2, 917 01 Trnava, Slovakia; 6 Stanford Prevention Research Center, Department of Medicine, Stanford University School of Medicine, Stanford, CA 94305, USA; laronica@stanford.edu; 7 Advanced Microscopy Facility, VBCF, Vienna Biocenter (VBC), 1030 Vienna, Austria; juraj.gregan@univie.ac.at

**Keywords:** Nrl1, pre-mRNA splicing, protein–protein interactions, phosphorylation, Cka1, fission yeast

## Abstract

Pre-mRNA splicing is a key process in the regulation of gene expression. In the fission yeast *Schizosaccharomyces pombe*, Nrl1 regulates splicing and expression of several genes and non-coding RNAs, and also suppresses the accumulation of R-loops. Here, we report analysis of interactions between Nrl1 and selected RNA-processing proteins and regulation of Nrl1 function by phosphorylation. Bacterial two-hybrid system (BACTH) assays revealed that the N-terminal region of Nrl1 is important for the interaction with ATP-dependent RNA helicase Mtl1 while the C-terminal region of Nrl1 is important for interactions with spliceosome components Ctr1, Ntr2, and Syf3. Consistent with this result, tandem affinity purification showed that Mtl1, but not Ctr1, Ntr2, or Syf3, co-purifies with the N-terminal region of Nrl1. Interestingly, mass-spectrometry analysis revealed that in addition to previously identified phosphorylation sites, Nrl1 is also phosphorylated on serines 86 and 112, and that Nrl1-TAP co-purifies with Cka1, the catalytic subunit of casein kinase 2. In vitro assay showed that Cka1 can phosphorylate bacterially expressed Nrl1 fragments. An analysis of non-phosphorylatable *nrl1* mutants revealed defects in gene expression and splicing consistent with the notion that phosphorylation is an important regulator of Nrl1 function. Taken together, our results provide insights into two mechanisms that are involved in the regulation of the spliceosome-associated factor Nrl1, namely domain-specific interactions between Nrl1 and RNA-processing proteins and post-translational modification of Nrl1 by phosphorylation.

## 1. Introduction

Eukaryotic cells use various mechanisms to regulate gene expression. Among these processes, pre-mRNA splicing plays an important role. pre-mRNA splicing is known as a process in which introns are removed from a pre-mRNA to create a mature RNA molecule. This highly orchestrated process is driven by a multimegadalton ribonucleoprotein complex known as a spliceosome, comprising of five main small nuclear ribonucleoproteins (snRNP), U1, U2, U4/U6, and U5, that associate with its cognate small nuclear RNA (snRNA), and of numerous splicing proteins and associated factors [1,2,3,4]. It is well known that during the splicing events the spliceosome dynamically changes its composition and structure. Since these changes are highly intricate and fine-tuned, they are also susceptible to many alterations. As such, mutations of splicing factors or alterations in the mechanisms regulating the splicing processes might lead to tumorigenesis or various developmental diseases [5,6,7,8,9].

Importantly, the function of many splicing factors and RNA binding proteins (RBPs) were found to be regulated by post-translational modifications [10]. Among these, phosphorylation plays a dominant role. Currently, several protein kinases, including SR protein kinases 1 and 2, Dsk1, Prp4, and Clk/Sty, are known to be involved in the regulation of pre-mRNA splicing [11,12,13,14,15]. Similarly, a fine balance between phosphorylation and dephosphorylation by both activation and inhibition of the PP1 and PP2A phosphatases has been shown to be required for splicing catalysis [16,17,18,19].

Recent studies have shown that the accurate assembly and catalytic activation of spliceosomal components SRSF1, PRP6, and PRP31 rely upon their phosphorylation [19,20,21]. Similarly, the dephosphorylation of U5 and U2 snRNP component was shown to be crucial for spliceosome activity [18,22]. Other studies found that phosphorylation of the splicing factors SF1, SRSF1, and SRSF3 also modulates interactomes of these factors [23,24,25,26]. Interestingly, it was shown that the SR proteins have to be hypo-phosphorylated to properly regulate the export of mature RNA molecules and translation, which further underlines the importance of the dynamic regulation of splicing processes [27,28,29,30,31,32]. Furthermore, it was found that phosphorylation is also important for the function of heterogeneous nuclear ribonucleoproteins (hnRNPs) and splicing factors belonging to other RBP families. As such, the phosphorylation of specific amino acid residues of hnRNP A1 was found to regulate the rate of its nuclear import, to modulate its strand annealing activity, and to facilitate the capping of the newly replicated telomeres [33,34,35]. Similarly, hnRNP M, which co-transcriptionally represses gene expression by influencing both constitutive and alternative splicing decisions, was shown to be regulated via phosphorylation, thus specifically controlling the intron removal in the innate immune-activated transcripts [36]. Moreover, the phosphorylation induced conformational remodeling of the polypyrimidine tract binding protein 2 (PTBP2) was shown, similar to remodeling of the unstructured serine arginine rich region of the SR proteins [37,38,39], to alter the protein–protein and protein–RNA interactions [40,41]. Altogether, these findings strongly point towards the importance of phosphorylation for the regulation of various factors involved in the processes of pre-mRNA splicing.

Recently, we have reported that the evolutionary conserved spliceosome-associated factor Nrl1 plays an important role in the regulation of splicing and expression of a subset of genes and non-coding RNAs in the fission yeast *S. pombe* [42]. Previously, it was shown that Nrl1 was part of a complex that cooperates with the spliceosome to target intron-containing precursor telomerase RNA and cryptic introns to facilitate splicing and production of short interfering RNAs at these loci [43]. Additionally, it was found that Nrl1 interacts with MTREC complex ATP-dependent RNA helicase Mtl1 and telomerase regulatory factor Ctr1, thus participating in the recognition and degradation of mis-spliced or unspliced RNA products [44].

In this work, we analyzed the biological significance of Nrl1 phosphorylation and studied the protein–protein interactions between Nrl1 and RNA-processing proteins that are known to associate with Nrl1. By generating specific truncated Nrl1 mutants, we identified regions that mediate the binding of Nrl1 to RNA helicase Mtl1 and splicing factors Ctr1, Ntr2, and Syf3. Importantly, our mass-spectrometry analyses revealed new Nrl1 phosphorylation sites and showed that Cka1 kinase co-purifies with Nrl1-TAP. Cka1 purified from *S. pombe* cells phosphorylated bacterially expressed Nrl1 fragments on two serine residues, raising the possibility that Cka1 is directly involved in phosphorylation of Nrl1. Moreover, non-phosphorylatable *nrl1* mutants showed defects in gene expression and splicing. This work reveals novel features involved in the regulation of the spliceosome-associated factor Nrl1 defined by its domain-specific interactions and post-translational modifications.

## 2. Results

### 2.1. Interaction Studies of Nrl1 and Selected RNA-Processing Factors Using a BACterial Two-Hybrid (BACTH) Assay

Defining the protein domains that mediate protein–protein interactions can help to reveal the molecular function of proteins within their protein complexes. Previously, we showed that the *S. pombe* protein Nrl1 is part of the spliceosome [42]. Results of various affinity purification strategies supported by the yeast two-hybrid assay suggested that Nrl1 might directly interact with several splicing factors [42,43,45]. However, the Nrl1 domains responsible for these interactions have not been identified.

**Figure 1:**
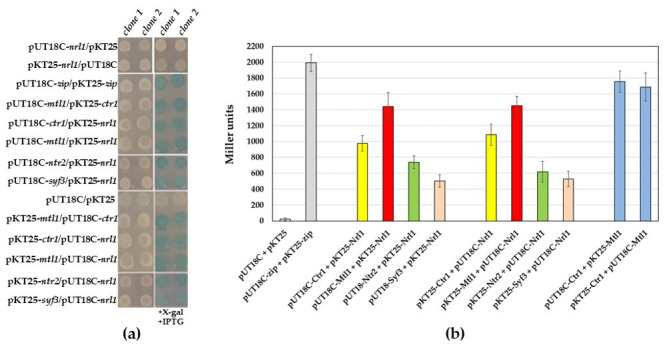
Physical interactions between Nrl1 and selected RNA-processing factors analyzed by the BACTH assay. (**a**) In vivo protein–protein interaction studies of Nrl1 and RNA-processing factors Ctr1, Ntr2, Syf3, and Mtl1 helicase. The *nrl1*, *ctr1*, *mtl1*, *ntr2*, and *syf3* were sub-cloned into the pUT18C and pKT25 plasmids. *E. coli* cells transformed with the indicated constructs were plated on standard LB agar plates and LB agar plates containing X-gal and IPTG. The pUT18-zip and pKT25-zip plasmids and interactions between the Ctr1 and Mtl1 were used as positive controls. As negative controls, the empty vectors pUT18C and pKT25 were used. (**b**) Intensities of interactions between the studied proteins were evaluated by β-Galactosidase activity assay. Quantification of β-galactosidase activities of transformants are shown in Miller units. Data represent mean values ± S.D. from eight independent biological replicates.

To decipher the principles of the protein–protein interactions of Nrl1, we have decided to identify Nrl1 domains that mediate its interactions with selected RNA-processing factors. In the first set of experiments, we used the bacterial two-hybrid (BACTH) assay and verified the protein–protein interactions of full length Nrl1 with known RNA-processing interactors Ctr1, Mtl1, Ntr2, and Syf3 (Figure 1a). We found that the strength of interaction between the Nrl1 and tested proteins decreased in the following order: Mtl1 > Ctr1 > Ntr2 > Syf3. Nrl1 interaction with Ctr1 produced 977.3 ± 89.5 and 1087.5 ± 134.4 Miller units in β-galactosidase assay when using *nrl1* and *ctr1* sub-cloned into pUT18C and pKT25 plasmids, respectively. In the case of *nrl1* and *mtl1* sub-cloned into pUT18C and pKT25 plasmids, respectively, an increment in Miller units 1439.0 ± 175.4 or 1451.7 ± 116.7 was observed. We detected lower values in Miller units for interactions of Nrl1 with Ntr2 and Syf3 when their alleles were sub-cloned into pUT18C and pKT25 plasmids, respectively (738.9 ± 80.2 and 467.4 ± 63.9 Miller units for interaction between Nrl1 and Ntr2, respectively, and 503.6 ± 77.8 and 529.4 ± 98.0 Miller units for interaction between Nrl1 and Syf3, respectively) (Figure 1b).

**Figure 2:**
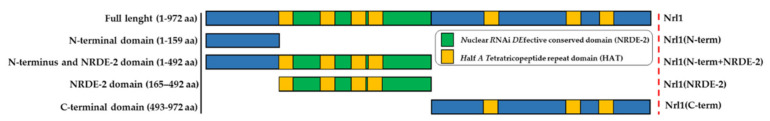
Schematic representation of known Nrl1 domains and motifs and the regions that were truncated.

To define Nrl1 domains mediating the interactions of Nrl1 with selected RNA-processing factors, we created *nrl1* truncations sub-cloned into the pUT18C plasmid. These constructs expressed the N-terminal region (1–159 aa), N-terminus with NRDE2 domain (1–492 aa), NRDE-2 domain (165–492 aa), and the C-terminal region (493–972 aa) of Nrl1 (Figure 2).

**Figure 3:**
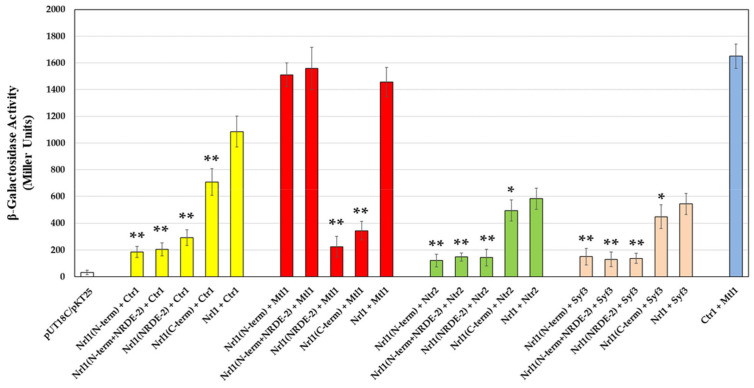
Physical interactions between wild-type Nrl1, truncated Nrl1 proteins, and RNA-processing factors Ctr1, Mtl1, Ntr2, and Syf3 analyzed by BACTH assay. The interaction between the Ctr1 and Mtl1 was used as a positive control. Data represent mean values ± S.D. from eight independent biological replicates. Statistical significance of truncated domain construct interactions compared to Nrl1 and selected RNA-processing factor interactions was determined using one-way analysis of variance (ANOVA) (*p*-values: *–*p* ≤ 0.05 and **–*p* ≤ 0.01).

The Nrl1 truncation constructs were further tested for the ability to interact with the selected RNA-processing factors that were sub-cloned into pKT25 plasmid. We observed similar intensities of interaction of the Nrl1(N-term) and the Nrl1(N-term + NRDE-2) constructs with Mtl1 (1511.2 ± 89.4 or 1558.2 ± 159.3 Miller units for the N-terminal region and the N-terminus with NRDE2 domain and Mtl1, respectively), as compared to Nrl1 and Mtl1 (1456.1 ± 109.3 Miller units). This suggests that the N-terminal region of Nrl1 is important for binding to Mtl1. The interactions of the Nrl1(NRDE-2) and the Nrl1(C-term) domain constructs with Mtl1 were significantly lower (223.6 ± 78.6 and 343.7 ± 69.1 Miller units, respectively). Unlike the Nrl1 and Mtl1 interaction, the interactions of Nrl1 with splicing factors were dependent on the presence of the C-terminal region of Nrl1. While the intensities of interactions between the splicing factor Ctr1 and the Nrl1(N-term), the Nrl1(N-term + NRDE-2), and the Nrl1(NRDE-2) constructs were in the range of 184.7 ± 41.6 to 291.5 ± 58.1 Miller units, the intensity of interaction between the construct containing the C-terminal region of Nrl1 (Nrl1(C-term)) and Ctr1 was closer to the intensity of interaction between Nrl1 and Ctr1 (708.5 ± 99.2 and 1085.0 ± 115.3 Miller units, respectively). Similarly, the intensities of interactions between the splicing factor Ntr2 or Syf3 and the Nrl1(N-term), the Nrl1(N-term + NRDE-2) and the Nrl1(NRDE-2) constructs were in the range of 121.1 ± 47.3 to 147.5 ± 62.1 Miller units or 130.2 ± 54.1 to 150.3 ± 61.6 Miller units, respectively. The intensities of interaction between the Nrl1(C-term) construct and Ntr2 or Syf3 were 494.7 ± 78.8 or 448.9 ± 88.7 Miller units, respectively. These values were similar to the intensity of interaction between Nrl1 and Ntr2 or Nrl1 and Syf3 (583.3 ± 79.4 or 544.8 ± 79.0 Miller units, respectively) (Figure 3, Supplementary Table S1). These findings are consistent with the notion that the N-terminal region of Nrl1 is important for the interaction between Nrl1 and Mtl1 while the C-terminus of Nrl1 mediates the interactions between Nrl1 and other splicing factors.

### 2.2. Analysis of Interactomes of Truncated Forms of Nrl1 by Tandem Affinity Purification

To validate the aforementioned findings on interactions between Nrl1 and selected RNA-processing factors, we expressed truncated versions of Nrl1 fused with the tandem affinity purification (TAP) tag in *S. pombe*. The TAP tag was inserted in-frame immediately after 477 nt or 1506 nt of *nrl1* thus allowing the expression of Nrl1 truncations Nrl1(1–159aa)-TAP (counterpart of Nrl1(N-term) construct used in the BACTH assay) and Nrl1(1–502aa)-TAP (counterpart of Nrl1(N-term + NRDE-2) construct used in the BACTH assay), respectively (Figure S1a). Proteins associated with Nrl1-TAP, Nrl1(1–159aa)-TAP and Nrl1(1–502aa)-TAP were isolated by tandem affinity purification and analyzed by mass-spectrometry.

We found that while the Nrl1-TAP co-purified with spliceosome proteins, its truncated forms had a significantly reduced ability to bind to spliceosome proteins. While Nrl1(1–159aa)-TAP co-purified only with the ATP-dependent RNA helicase Mtl1, the Nrl1(1–502aa)-TAP co-purified with Mtl1 and the ubiquitin-protein ligase E4 Ppr19 (Figure 4, Supplementary Table S2). These findings suggest that the N-terminal region of Nrl1 is important for the interaction with Mtl1 and reveal a possible role of the NRDE2 domain in interaction with Prp19. Interestingly, the Nrl1(1–502aa)-TAP, in addition to RNA-processing factors Mtl1 and Prp19, co-purified with proteins that bind small nuclear RNAs (Utp14, Nhp2) [46] and SAGA complex proteins (Taf5, Taf6, Taf9 and Taf12), which mediate nucleosomal histone acetyltransferase activity and are thought to help recruit the transcription machinery [47,48] (Figure 4, Supplementary Table S2). The findings that the interactome of the full length Nrl1 protein contains numerous spliceosomal factors, but interactomes of Nrl1 truncated forms counts only a few of these factors suggest that C-terminal region of Nrl1 protein has an important role in mediating and stabilizing the binding of Nrl1 into the spliceosome. This is consistent with our model suggesting the C-terminal region of the Nrl1 protein to be important for its stable binding with the spliceosome.

### 2.3. Analysis of Nrl1 Phosphorylation

**Figure 5:**
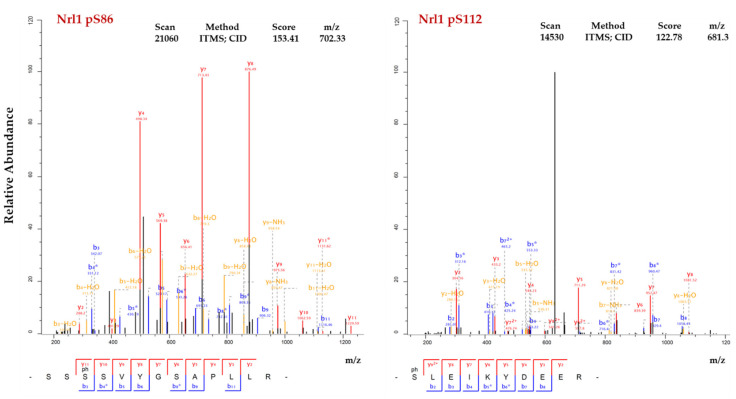
Fragmentation (MS/MS) spectra of newly identified Nrl1 phosphopeptides. Annotated fragmentation spectrum of a singly phosphorylated Nrl1 peptide (SSpSSVGSAPLLR) containing pS86 and a singly phosphorylated Nrl1 peptide (pSLEIKYDEER) containing pS122. Overall fragment coverage is indicated in the peptide sequences below the spectra with the b-ions in blue and y-ions in red. The asterisks in the spectra (*) denote peptide fragments with a neutral loss of H_3_O_4_P (97.98 Da) originating from the corresponding phosphorylated fragment ions.

Previous studies have shown that Nrl1 is phosphorylated on several amino acid residues [42,45,49,50,51,52,53]. This suggested that, similarly to other splicing factors, the function of Nrl1 might be regulated by phosphorylation. To find out if there are any additional phosphorylations of Nrl1 that have been missed in the previous studies, we mapped the Nrl1 phosphorylation sites of Nrl1-TAP by mass spectrometry. We found that in addition to previously identified phospho-sites (T26, S122, S131, and S970), Nrl1 is also phosphorylated on S86 and S112 (Figure 5, Supplementary Table S3).

### 2.4. In Vitro Kinase Assay to Assess Nrl1 Phosphorylation by Cka1

**Figure 6:**
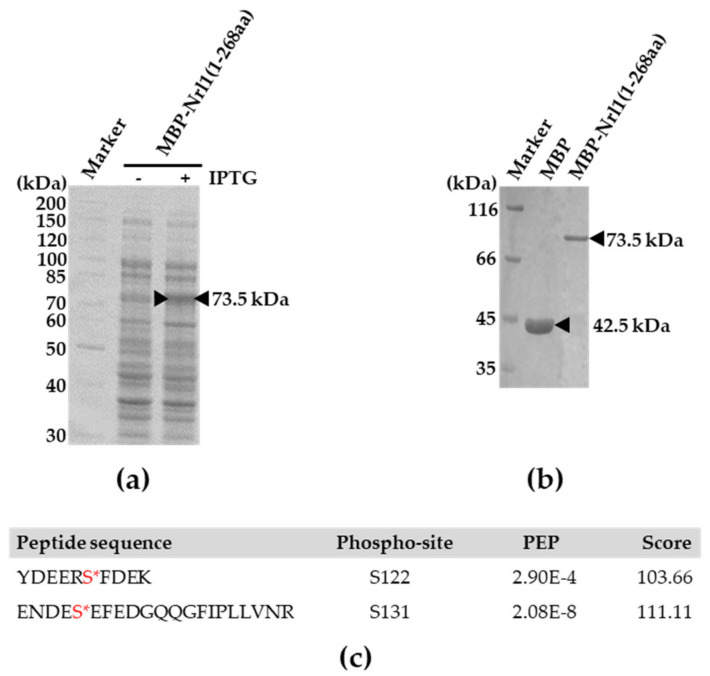
Expression, purification, and in vitro phosphorylation of Nrl1 fragment by Cka1 protein kinase. (**a**) Nrl1 protein fragment (1–268aa) was expressed in fusion with maltose binding protein (MBP) in *E. coli.* (**b**) MBP-Nrl1(1–268aa) protein was affinity purified using amylose resin. As a control of purification efficiency, MBP was used. (**c**) MBP-Nrl1(1–268aa) purified from *E. coli* was incubated with Cka1-TAP purified from *S. pombe* cells and analyzed by mass spectrometry. Two Nrl1 serine residues that were found to be phosphorylated are marked by asterisks and highlighted in red.

Our finding that Cka1 protein kinase co-purifies with the Nrl1-TAP as a part of the spliceosome (Figure 4 and Supplementary Table S2) together with the fact that Nrl1 amino acid residues S86, S112, S122, and S131 match the casein kinase II consensus phosphorylation motifs [S/T-X-X-E/D, S/T-X-E/D, S/D and their variations] (Table S3) suggest that Cka1 may regulate the function of Nrl1 through its direct phosphorylation. To test this, we performed an in vitro kinase assay with a bacterially expressed fragment of Nrl1 and Cka1-TAP purified from cycling *S. pombe* cells. The Nrl1(1–268aa) fragment that accommodates five out of six Nrl1 phosphorylation sites was expressed as a fusion with maltose-binding protein (MBP) (Figure 6a). The MBP-Nrl1 fragment (~73.5 kDa) was affinity purified using the amylose resin (Figure 6b) and subsequently used for in vitro kinase assay with the Cka1-TAP. To determine the site(s) of phosphorylation, the reaction mixture was directly subjected to mass spectrometry analysis. We found that Nrl1 was phosphorylated at serine residues S122 and S131 (Figure 6c, Table S4). Although this phosphorylation is likely due to Cka1 activity, we cannot exclude the possibility that other kinases are present as contaminants in our Cka1-TAP purification (e.g., Nak1) phosphorylate Nrl1.

### 2.5. Splicing Function of Nrl1 Is Regulated by Phosphorylation

Previously we have shown that *nrl1*Δ cells display significant changes in expression and splicing of a subset of genes and non-coding RNAs [42]. To determine whether the function of Nrl1 is regulated by phosphorylation, we generated *nrl1*^S/A^ mutants where we mutated the serine residues found to be phosphorylated to alanine, which can no longer be phosphorylated (*nrl1*^S86A/S112A^, *nrl1*^S122A/S131A^, *nrl1*^S86A/S112A/S122A/S131A^ and *nrl1*^S86A/S112A/S122A/S131A/S970A^) and corresponding phosphomimetic *nrl1*^S/D^ mutants carrying serine to aspartate substitutions (*nrl1*^S86D/S112D^, *nrl1*^S122D/S131D^, *nrl1*^S86D/S112D/S122D/S131D^ and *nrl1*^S86D/S112D/S122D/S131D/S970D^) (Table S5). Expression and stability of non-phosphorylatable and phosphomimetic Nrl1 proteins were confirmed by Western blotting (Figure S1b).

**Figure 7:**
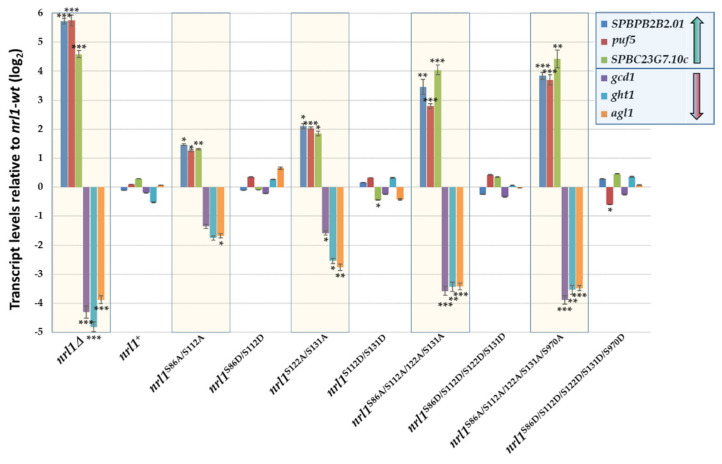
Analysis of gene expression in *nrl1*Δ and phosphorylation-site mutants *nrl1*^S/A^ and *nrl1*^S/D^. RNA was isolated from cells in the exponential phase (OD_600_ = 0.5–0.6), and gene expression of *SPBPB2B2.01*, *puf5*, *SPBC23G7.10c*, *gcd1*, *ght1*, and *agl1* was analyzed using qPCR. The data represent mean values ± S.D. of transcript levels relative to wild-type *nrl1*^+^ after normalization to *act1* from four independent biological replicates. Statistical significance in expression of studied genes in *nrl1* mutants compared to wild-type *nrl1*^+^ was determined using one-way analysis of variance using ANOVA (*p*-values: *—*p* ≤ 0.05, **—*p* ≤ 0.01, ***—*p* ≤ 0.001).

In order to study the importance of Nrl1 phosphorylation for the processes regulating the gene expression, we selected three representative genes that were shown to be upregulated (*SPBPB2B2.01*, *puf5* and *SPBC23G7.10c*) and three representative genes that were shown to be downregulated (*gcd1*, *ght1* and *agl1*) in *nrl1*Δ mutant [42]. To check the impact of Nrl1 phosphorylation-site mutations on expression of these genes, we assayed the changes in their transcript levels in *nrl1*Δ, *nrl1*^S/A^, and *nrl1*^S/D^ mutants using qPCR. Consistent with our RNA-seq data [42], we found that in *nrl1*Δ mutant the expression levels of *SPBPB2B2.01*, *puf5*, and *SPBC23G7.10c* were substantially increased and the expression levels of *gcd1, ght1*, and *agl1* were decreased. Interestingly, we found that *nrl1*^S/A^ mutants resembled the changes detected in *nrl1*Δ mutant. In contrast, the transcript levels of analyzed genes in the phosphomimetic *nrl1*^S/D^ mutants were not significantly affected (except of expression of *SPBC23G7.10c* in *nrl1*^S122D/S131D^ and *puf5* in *nrl1*^S86D/S112D/S122D/S131D/S970D^) and were similar to those of wild type *nrl1*^+^ cells (Figure 7). The finding that expression of analyzed genes is affected in non-phosphorylatable *nrl1*^S/A^ mutants but stayed mostly normal in phosphomimetic *nrl1*^S/D^ mutants suggested that the function of Nrl1 is regulated by phosphorylation.

**Figure 8:**
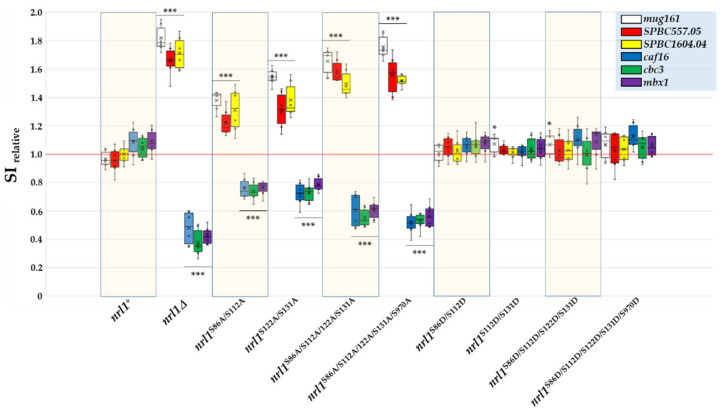
Analysis of splicing efficiency in *nrl1*Δ and phosphorylation-site mutants *nrl1*^S/A^ and *nrl1*^S/D^. The relative splice-index (SI relative) is shown for the 3rd intron of *mug161* and 1^st^ introns of *SPBC557.05*, *SPBC1604.04*, *caf16*, *cbc3*, and *mbx1*. The data represent mean values ± S.D. of splicing index (SI) relative to wild-type from three independent biological replicates. Statistical significance in splicing of studied genes in *nrl1* mutants compared to wild-type *nrl1*^+^ was determined using one-way analysis of variance (ANOVA) (*p*-values: *–*p* ≤ 0.05 and ***–*p* ≤ 0.001).

The finding that non-phosphorylatable *nrl1*^S/A^ mutants exhibit dysregulated gene expression prompted us to check the impact of phosphorylation-site mutations of Nrl1 on the efficiency of pre-mRNA splicing. To test this, we selected representative genes in which splicing was shown to be altered in *nrl1*Δ mutant [42]. We found that similar to the *nrl1*Δ mutant, the non-phosphorylatable *nrl1*^S86A/S112A^, *nrl1*^S122A/S131A^, *nrl1*^S86A/S112A/S122A/S131A^, and *nrl1*^S86A/S112A/S122A/S131A/S970A^ mutants suffered from the decreased efficiency of splicing of *mug161*, *SPBC557.05*, and *SPBC1604.04* (SI > 1), while the splicing efficiency of *caf16*, *cbc3*, and *mbx1* was increased (SI < 1) in these mutants, as compared to wild type *nrl1*^+^ cells. In the case of phosphorylation mimicking *nrl1*^S/D^ mutants, the splice-index profiles of the analyzed genes were mostly similar to those of wild type *nrl1*^+^ cells (except of *SPAC1F3.09* in *nrl1*^S122A/S131A^ and *nrl1*^S86A/S112A/S122A/S131A^ mutants) (Figure 8).

Collectively, the analyses of gene expression and splicing efficiency of generated phosphomutants of Nrl1 indicated that phosphorylation plays an important role in the regulation of the biological function of the Nrl1 protein.

## 3. Discussion

*S. pombe* Nrl1 is a protein of about 113 kDa that contains, similarly to its *C. elegans* and human NRDE-2 orthologues, several half-a-tetratricopeptide repeat domains (HATs). HATs can be found in RNA-binding proteins and are often involved in protein–protein interactions [54]. Nrl1 contains seven HATs, which are distributed within the centrally positioned NRDE-2 domain (four HATs) and the C-terminal region of Nrl1 (three HATs). However, contrary to the N-terminal region of the *ce*NRDE-2, which contains a defined arginine-serine (RS) rich domain [55], the N-terminal part of Nrl1 has no defined RS domain. Instead, it is comprised of seven disordered regions (1–59 aa, 65–66 aa, 69–77aa, 81–85 aa, 118–121 aa, 124–131 aa, and 144–145 aa) containing four individual arginine-serine pairs (33–34 aa, 52–53 aa, 83–84 aa, and 121–122 aa). In addition, several studies have found that the N-terminus of Nrl1 is phosphorylated [42,50,51,52].

In contrast to *ce*NRDE-2, which is associated with the RNAi machinery and is necessary for both tri-methylation of H3K9 at genomic loci targeted by siRNAs and for the inhibition of transcription elongation downstream of the siRNA-targeted sequences [55,56], the Nrl1 is not involved in the RNAi pathway [42]. Instead, Nrl1 secures genome stability by preventing the accumulation of R-loops and promoting DNA repair of these structures through homologous recombination [42]. Interestingly, unlike the *S. pombe nrl1*Δ mutant, DSBs that accumulate after depletion of *hs*NRDE-2 do not directly rely on the accumulation of R-loops, but significantly affect R-loop profiles at specific loci, suggesting an independent connection of *hs*NRDE-2 with transcriptional regulation through formation/resolution of the R-loops [57]. Importantly, recent studies identified Nrl1 as a spliceosome-associated factor that interacts with many factors involved in RNA biogenesis [42,43,44,45].

In this work, for the first time, we describe region-specific interactions of Nrl1 with selected RNA-processing factors and show that the phosphorylation of Nrl1, possibly by Cka1 protein kinase, may be involved in the proper expression and efficient splicing of a subset of genes.

To determine the domain specific interactions of Nrl1 with RNA-processing factors, we generated Nrl1 truncation constructs containing its N-terminal region, NRDE-2 domain or C-terminal region (Figure 2). Using BACTH assay, we found that ATP-dependent RNA helicase Mtl1 binds to the N-terminal region of Nrl1 (Figure 3 and Figure 4). Interestingly, the N-terminal region of Nrl1 formed a stable sub-module with the Mtl1 helicase but not with other splicing factors tested. This suggests that the N-terminal region of Nrl1 functions as a scaffold domain that recruits Mtl1 to the spliceosome. We could speculate that when Nrl1-Mtl1 is anchored to the spliceosome, then Ctr1 might be attracted and Nrl1-Mtl1-Ctr1 complex might help to control the degradation of unspliced pre-mRNA [43,44]. On the other hand, by analyzing the interactome of the NRDE-2 domain, we detected weak interactions of this domain with Utp14 and Nhp2 (snoRNA binding proteins) and with Taf5, Taf6, Taf9, and Taf12 (SAGA complex components). SAGA complex is thought to mediate nucleosomal histone acetyltransferase activity and help the recruitment of the basal transcription machinery [47,48]. Regarding the Utp14 and Nhp2 interactors, these are known to be involved in the co-transcriptional assembly of specific proteins on H/ACA box snoRNAs, thus helping to mediate the exchange of factors during snoRNA processing [46]. Our findings that NRDE-2 domain of Nrl1 interacts with proteins of SAGA complex and snoRNA binding proteins might suggest possible but probably redundant affinity of this domain to these proteins. It is also tempting to speculate that deletion of C-terminal region of Nrl1 induces the conformational changes in NRDE-2 domain of truncated Nrl1(1–502aa)-TAP, thus modulating and mediating the binding of proteins of SAGA complex and snoRNA binding proteins. Finally, by analyzing the C-terminal part of Nrl1, we found that this region is important for Nrl1 interactions with splicing factors, which is likely to secure stable anchoring of Nrl1 to the spliceosome (Figure 1 and Figure 4).

Previous studies have shown that many splicing factors have to be phosphorylated in order to function properly [8,11,12,13,14,15,16,17,18,19,20,21,22,23,24,25,26,27,28,29,30,31,32,33,34,35,36,37,38,39,40,41]. Although previous studies showed that Nrl1 is phosphorylated on multiple sites (T26, S122, S131 and S970) [49,50,51,52,53], little was known about the importance of these post-translational modifications. Here, by analyzing the isolated Nrl1 complexes we found that Nrl1 is also phosphorylated on residues S86 and S112 (Figure 5). It is noteworthy that phosphorylated residues S86 and S122 are part of the sequences containing the individual arginine-serine (RS) pairs located in the N-terminal region of Nrl1. Dynamic phosphorylation of splicing factors, mainly at the serine residues within their RS domains, is known to be important for the proper splicing function of these factors [25,28,57,58,59,60]. Our in silico analysis suggested that Nrl1 residues S86, S112, S122, and S131 might be recognized and phosphorylated by members of the casein kinase II family (CKII) (Supplementary Table S3). Moreover, our findings that Cka1 protein kinase co-purifies with Nrl1-TAP (Figure 4, Supplementary Table S2) and that at least two Nrl1 residues, S122 and S131, are phosphorylated in vitro in the presence of Cka1 (Figure 6c, Supplementary Table S4) raised the possibility that Nrl1 might be a direct target of Cka1 kinase. Interestingly, a recent study by Yan et al. identified Cka1 protein kinase to be a part of the spliceosomal complex of the *S. pombe* [61]. Although they did not detect its direct interactors, their findings suggest that this kinase might be an important regulator of several splicing proteins including Nrl1. In this respect, it would be interesting to characterize in detail the phosphoproteome of Cka1 and identify protein kinases that phosphorylate individual Nrl1 phospho-sites.

Interestingly, mutating phosphorylated Nrl1 residues to non-phosphorylatable or phosphorylation mimicking residues, followed by the analysis of generated mutants, revealed that phosphorylation of Nrl1 may affect its function. Gene expression analysis and measurements of splicing efficiency propose that phosphorylation of N-terminal region of Nrl1 on residues S86, S112, S122, and S131 is required for the proper expression and splicing of a subset of analyzed genes. This raises the possibility that phosphorylation of Nrl1 N-terminus may play an important role in the regulation of Nrl1 function. Interestingly, the finding that the region of Nrl1 which mediates interaction with Mtl1 is phosphorylated, possibly by Cka1, opens the possibility that interaction of Nrl1 with RNA-processing factors might be dynamically regulated through phosphorylation. Previously, several studies showed the protein–protein interactions and spliceosomal related functions of splicing factors to be partly or entirely dependent on phosphorylation [24,62,63,64,65]. Further studies are needed to address the questions about the possible role of Nrl1 phosphorylation in the regulation of protein–protein interactions.

**Figure 9:**
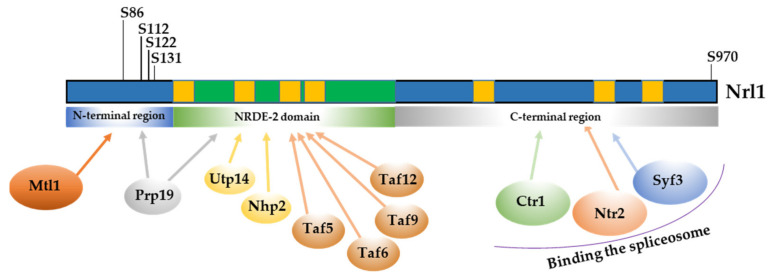
Schematic representation of the identified Nrl1 phosphorylation and domain-specific interactions of Nrl1 protein with various RNA-processing factors.

In summary, our findings provide new insights into two mechanisms by which Nrl1 is regulated: domain/region specific protein–protein interactions and post-translational modification by phosphorylation. Our results are consistent with the notion that the N-terminal part of Nrl1 mediates interaction with Mtl1 helicase while the C-terminal part secures anchoring of Nrl1 to the spliceosome through binding with other splicing factors. Our analysis of post-translational modifications further reveals that Nrl1 phosphorylation within its N-terminal region, possibly mediated by the Cka1 protein kinase, is important for proper function of Nrl1 (Figure 9). In future studies, it will be interesting to investigate the possibility that these two mechanisms are interconnected and to find out if their mutual cooperation is necessary to regulate the function of Nrl1.

## 4. Materials and Methods

### 4.1. Strains, Media and Primers

The genotypes of the strains and the sequences of primers used in this study are listed in Tables S6 and S7. Strains carrying a deletion or TAP tag have been constructed as described previously [66,67]. Rich YE+5S media (5.0 g/L yeast extract, 3.0% glucose, 0.1 g/L L-leucine, 0.1 g/L L-lysine hydrochloride, 0.1 g/L L-histidine, 0.1 g/L uracil, and 0.15 g/L adenine sulfate) was used to grow *S. pombe* strains. If necessary, 0.15 g/L G418, 0.1 g/L nourseothricin, or 0.2 g/L hygromycin B were added. *S. pombe* was transformed using the lithium acetate method [66].

### 4.2. BACterial Two-Hybrid (BACTH) Assay

**Figure 10:**
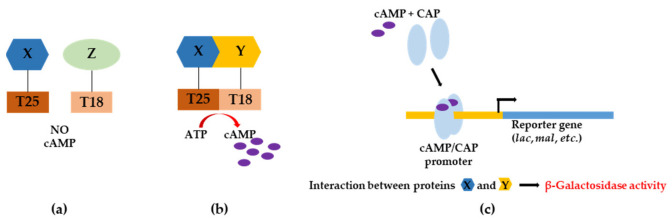
Scheme illustrating the principle of the BACTH assay. (**a**) Proteins T25-X and T18-Z that do not interact will not reconstitute adenylate cyclase activity. (**b**) Proteins T25-X and T18-Z that interact will reconstitute adenylate cyclase activity leading to production of cAMP. (**c**) Cyclic adenosine monophosphate (cAMP) bound to catabolite activator protein (CAP) positively regulates β-galactosidase expression.

The sequences encoding the ORFs of Nrl1 or selected RNA-processing factors and sequences encoding particular regions of Nrl1 (Figure 2) were PCR amplified using specific primers (Table S7), and sub-cloned in-frame with the T18 and T25 fragments of adenylate cyclase in pUT18C and pKT25 vectors of BACTH assay (Figure 10).

The created plasmid constructs were propagated in *E. coli* DH5 strain, purified with GeneJET Plasmid Miniprep Kit (ThermoFisher Scientific, Vilnius, Lithuania) and co-transformed into *E. coli* strain BTH101 (2.5 ng of each plasmid). Positive transformants were screened on LB plates supplemented with 40 µg/mL X-gal, 0.5 mM IPTG, 100 µg/mL ampicillin and 50 µg/mL kanamycin (at 37 °C). Transformants (about 100–200 colonies per plate) were replica plated on M63 minimal medium supplemented with 0.2% maltose, 40 µg/mL X-gal, 0.5 mM IPTG, 50 µg/mL ampicillin, and 25 µg/mL kanamycin, and incubated for 24 h at 37 °C. Positive (blue) transformants were cultured to stationary phase in LB media, spotted onto LB plates supplemented with 40 µg/mL X-gal, 0.5 mM IPTG, 100 µg/mL ampicillin, and 50 µg/mL kanamycin, and incubated for 24 h at 30 °C [68]. Intensities of interactions between studied proteins were evaluated by β-Galactosidase activity assay.

### 4.3. β-Galactosidase Activity Assay

β-Galactosidase activity assay was performed at 28 °C on stationary-phase aliquots of cultures in 3 mL of LB broth in the presence of 0.5 mM IPTG and antibiotics as described previously [68,69]. Optical density (OD_600_) of each culture was recorded. Cells were permeabilized by adding 15 µL of toluene and 15 µL of 0.1% SDS solution to 1.25 mL of culture, vortexed, plugged with cotton, and vigorously agitated for 40 min at 37 °C. Aliquots of 0.1 mL of the permeabilized cells were mixed with 0.9 mL of PM2 assay buffer and incubated for 5 min at 28 °C. PM2 assay buffer served as a blank. Then, 250 µL of 0.4% ONPG was added, and samples were incubated for 5 min at 28 °C. OD_420_ and OD_500_ were measured with a multiplate reader (xMark, Bio-Rad, Tokyo, Japan). β-Galactosidase activity was expressed as Miller units using the following equation:

Miller Units = 1000 × [(OD_420_ − 1.75 × OD_550_)]/(t × V × OD_600_),

where OD_420_ is the absorbance of the yellow o-nitrophenol, OD_550_ is the scatter from cell debris, which, when multiplied by 1.75 approximates the scatter observed at 420 nm, OD_600_ reflects cell density, t is reaction time in min and V is a volume of culture assayed in ml. The Miller Units give the change in OD_420_/min/mL of cells/OD_600_.

### 4.4. Western Blotting

*S. pombe* cells were grown in complete YE+5S medium at 32 °C. Expression of TAP-tagged proteins was confirmed by immunoblotting using PAP antibody (rabbit anti-peroxidase antibody linked to peroxidase, Dako, Japan) at 1:20,000 dilution (2% skim milk in 0.05% PBS-T). Tubulin was detected using mouse anti-α-tubulin mAb TAT1 at 1:1000 dilution (a gift from K. Gull, University of Oxford, Oxford, UK) followed by the secondary antibody anti-mouse IgG at 1:5000 dilution (A3562, Sigma, St. Luis, MO, USA). Enhanced Pierce ECL Western Blotting Substrate (32209, Thermo Scientific, IL, USA) and Amersham Hyperfilm^TM^ ECL (GE Healthcare Limited, Buckinghamshire, UK) were used for detection.

### 4.5. Tandem Affinity Purification

Twelve-liter cultures of wild type strain or strains expressing TAP-tagged versions of Nrl1 protein were grown to mid-log phase (OD_600_ ~ 0.8), and cells were collected by centrifugation. Yeast cell powders (50 g) were made from frozen cell pellets using SPEX SamplePrep 6770 Freezer/Mill SPEX (SamplePrep, NJ, USA) cooled by liquid nitrogen. Proteins were extracted using IPP150 buffer (50 mM Tris pH 8.0, 150 mM NaCl, 10% glycerol, 0.1% NP-40, supplemented with complete protease and phosphatase inhibitors and 1 mM PMSF). A total of 0.5 mL of IgG Sepharose™ 6 Fast Flow beads per sample (GE Healthcare, IL, USA) was washed with IPP150 buffer, mixed with protein extracts, and rotated for 2 h at 4 °C. The beads were washed with 10 bead volumes of IPP150 buffer and with 5 bead volumes of TEV cleavage buffer (TCB, 10 mM Tris pH 8.0, 150 mM NaCl, 10% glycerol, 0.1% NP-40, 0.5 mM EDTA and 1 mM DTT). Cleavage step was performed in 2 mL of TCB buffer supplemented with 400 Units of Turbo TEV protease (MoBiTec, Goettingen, Germany) for 2 h at 16 °C. 2 mL of eluates was supplemented with 6 µL of 1 M CaCl_2_ and mixed with 6 mL of Calmodulin binding buffer 1 (CBB1, 10 mM Tris pH 8.0, 150 mM NaCl, 10% glycerol, 0.1% NP-40, 1 mM imidazole, 1 mM Mg-Acetate, 2 mM CaCl_2_ and 10 mM β-mercaptoethanol). An amount totaling 0.15 mL of Calmodulin Sepharose™ 4B beads per sample (GE Healthcare, IL, USA) was washed with CBB1 buffer, added to a mixture of eluates and CBB1 buffer and incubated for 2 h at 4 °C. The beads were washed with 10 bead volumes of CBB1 and 5 bead volumes of Calmodulin binding buffer 2 (CBB2, 10 mM Tris pH 8.0, 150 mM NaCl, 1 mM Mg-Acetate, 2 mM CaCl_2_ and 1 mM β-mercaptoethanol). The proteins were step-eluted using bead volume of elution buffer (EB, 10 mM Tris pH 8.0, 150 mM NaCl, 1 mM Mg-acetate, 2 mM EGTA and 1 mM β-mercaptoethanol). Eluates from peak fractions were submitted for LC-MS/MS analysis.

### 4.6. LC-MS/MS Analysis

Samples were reduced in the presence of 5 mM DTT (30 min, 60 °C), alkylated by the addition of 15 mM iodoacetamide (20 min, RT/in dark) and the alkylation reaction was quenched by an additional 5 mM DTT. A total of 0.5 µg of modified sequencing grade trypsin (Promega, WI, USA), 1 mM CaCl_2_, and 1 mM phosphatase inhibitor mix (β-glycerophosphate, Na_3_VO_4_, KF, and disodium diphosphate) was added to the protein mixture, and the samples were incubated overnight at 37 °C. The reaction mixture was acidified by the addition of 0.5% TFA, while the peptides were purified by microtip C18 SPE and dried by vacuum centrifugation. For LC-MS analysis, a set of nanotrap columns (PepMap100 C18, 300 μm i.d. × 5 mm, 5 µm particle, size, Dionex, CA, USA) and nanoseparation columns (Acclaim PepMap 100 C18, 75 μm × 500 mm, Thermo Fisher Scientific, MA, USA) attached to an UltiMate 3000 RSLCnano system (Dionex, CA, USA) were used. The peptides were separated in 1 h gradient from 3% to 43% B with two mobile phases used: 0.1% FA (*v/v*) (A) and 80% ACN (*v/v*) with 0.1% FA (B). Spectral datasets were collected by Orbitrap Elite mass spectrometer (ThermoScientific, MA, USA) operating in the data-dependent mode using Top15 strategy for the selection of precursor ions for the HCD or CID fragmentation. Each of the samples was analyzed in two technical replicates. Obtained datasets were processed by MaxQuant (version 1.6.17.0) [70] with built-in Andromeda search engine using carbamidomethylation (C) as a permanent modification and oxidation (M) and phosphorylation (STY) as variable modifications. The search was performed against the *S. pombe* protein database (UniProt, downloaded 9.7.2019). The mass spectrometry proteomics data have been deposited in the ProteomeXchange Consortium via the PRIDE [71] partner repository with the dataset identifier PXD026669.

### 4.7. Expression and Purification of MBP-Nrl1(1-268aa) Fragment

*S. pombe nrl1* fragment (1804 nt) was PCR amplified with primers *nrl1exp_1-268_fw* (5′-ATTCTAGAATGCCGTCTAATCATAACACG-3′ (XbaI site is underlined)) and *nrl1exp_1-268_rv* (5′-ATCTGCAGTTATTAACCTGGATGCTCAATCAATAC-3′ (PstI site is underlined)), digested with XbaI and PstI and cloned into pMAL-TEV/6xHIS vector creating in-frame fusion to the carboxyl terminus of maltose binding protein (MBP). The MBP-Nrl1(1-268aa) fusion protein was expressed in *E. coli* (BL21(DE3)), extracted, bound to maltose-binding beads, and eluted with maltose according to the manufacturer’s instructions (New England Biolabs, MA, USA). The eluted fusion protein was concentrated and washed with kinase buffer (50 mM Tris-HCl (pH 7.5), 10 mM MgCl_2_, 5 mM DTT) by centrifugation using the Centricon-30 system and stored at −80 °C.

### 4.8. In Vitro Kinase Assay

The MBP-Nrl1(1-268aa) fusion protein (∼500 ng) was mixed with Cka1 protein kinase complex (20 µL) purified by tandem affinity purification from exponentially growing *S. pombe* cells (OD_600_ = 0.6). The mixture of MBP-Nrl1(1–268 aa) and Cka1 was incubated in 80 µL of kinase buffer containing 200 µM ATP at 30 °C for 2 h. After reaction, the mixture was snap frozen using liquid nitrogen. The phosphorylation of MBP-Nrl1(1-268aa) fragment was analyzed by mass-spectrometry.

### 4.9. RNA Isolation and RT-qPCR Analysis

Total RNA was isolated from cells growing in YE+5S media at 32 °C to the exponential phase (OD_600_ = 0.5−0.6) as described previously [72]. cDNA was prepared from 2 μg of the total RNA using the RevertAid First Strand cDNA Synthesis Kit (ThermoScientific, MA, USA) with random hexamer primers according to the manufacturer’s instructions. For qPCR, PowerUp™ SYBR® Green Master Mix (Thermo Fisher Scientific, MA, USA) was used as instructed. RT-qPCR was performed using CFX96TM Real-Time System (Bio-Rad, CA, USA). Primers used for measuring gene expression and splicing efficiency are listed in Table S7. Primer pairs were designed to specifically amplify either the spliced or the unspliced transcripts of the studied genes. Three to four biological replicates were analyzed for each gene, and *act1* was used as a reference control. Relative transcript levels or pre-mRNA and mRNA quantities were calculated by the ΔΔCt method [73].

### 4.10. Statistical Test for Significance

Statistical significance, denoted by *p* values less than 0.05, 0.01 and 0.001, was determined using one-way analysis of variance (ANOVA) tests.

## Figures and Tables

**Figure 4:** Proteins co-purifying with TAP-tagged Nrl1 and its truncated forms Nrl1(1–159aa) and Nrl1(1–502aa). RNA-processing factors analyzed for their interactions with Nrl1 protein using BACTH assay (see Figure 1) are highlighted in grey. Common contaminants such as ribosomal proteins and proteins identified in mock purification are excluded from this list. All identified proteins are listed in Supplementary Table S2.

**Table:**
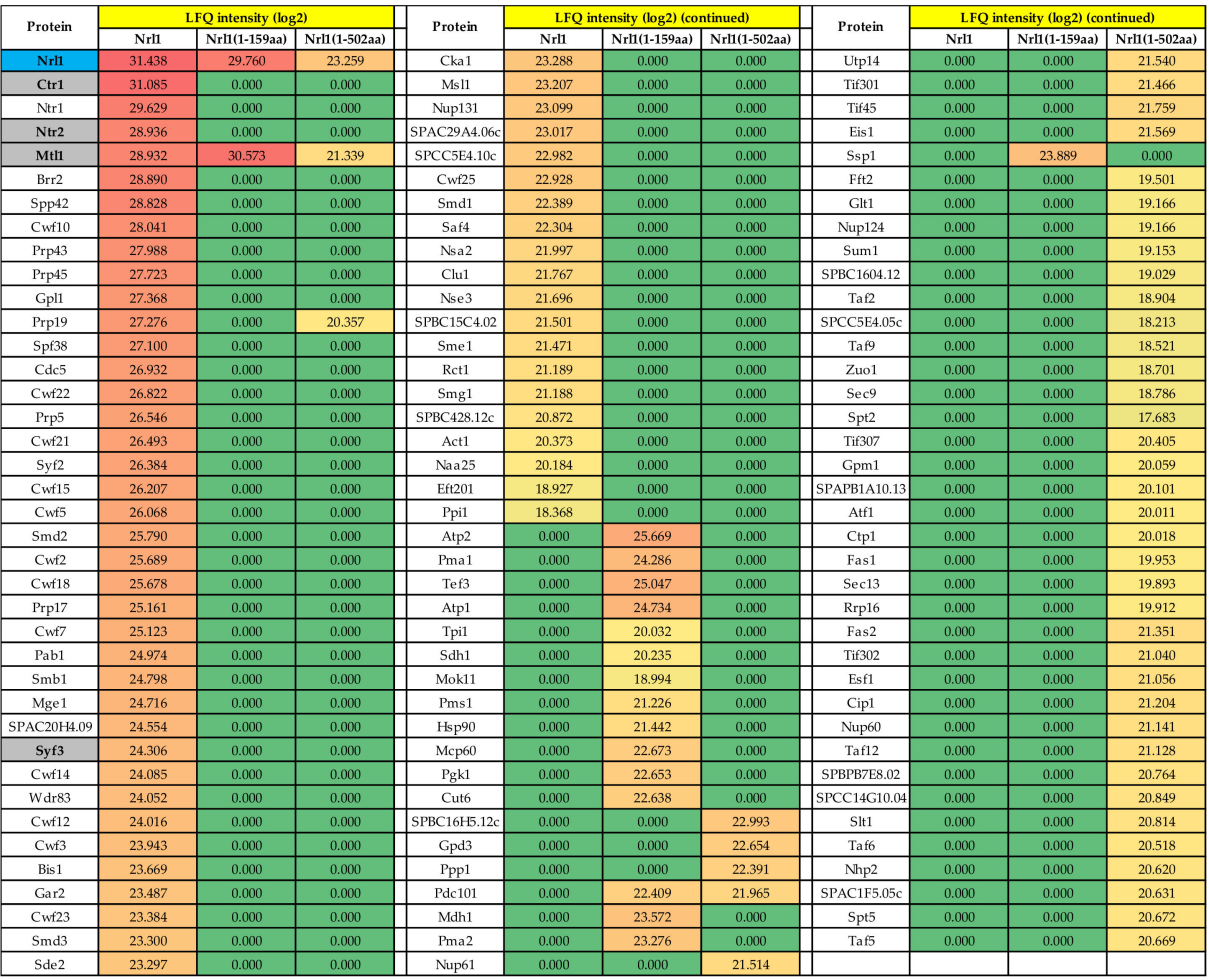


## Data Availability

The mass spectrometry proteomics data have been deposited in the ProteomeXchange Consortium via the PRIDE [71] partner repository with the dataset identifier PXD026669.

## Supplementary Materials

The following are available online at https://www.mdpi.com/article/10.3390/ijms22137011/s1.
