# Supplementary material for: Identification of Nrl1 Domains Responsible for Interactions with RNA-Processing Factors and Regulation of Nrl1 Function by Phosphorylation
Source: Int J Mol Sci. 2021 Jun 29;22(13):7011. doi: 10.3390/ijms22137011 (PMC8268110; doi:10.3390/ijms22137011)
Supplement: Supplementary file 1 [file ijms-22-07011-s001.zip › Figure S1.pptx]

## Slide 1
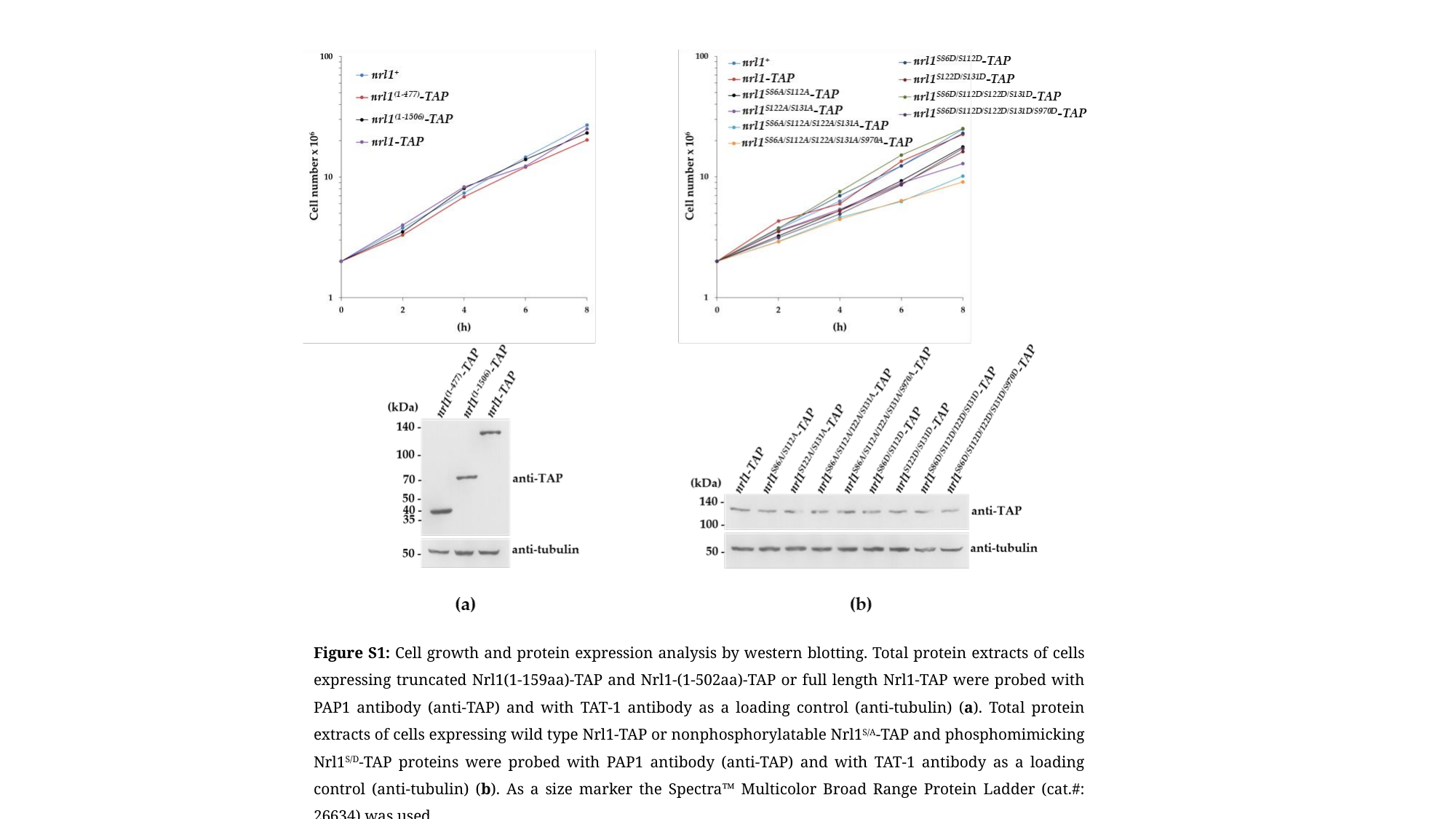

Figure S1: Cell growth and protein expression analysis by western blotting. Total protein extracts of cells expressing truncated Nrl1(1-159aa)-TAP and Nrl1-(1-502aa)-TAP or full length Nrl1-TAP were probed with PAP1 antibody (anti-TAP) and with TAT-1 antibody as a loading control (anti-tubulin) (a). Total protein extracts of cells expressing wild type Nrl1-TAP or nonphosphorylatable Nrl1S/A-TAP and phosphomimicking Nrl1S/D-TAP proteins were probed with PAP1 antibody (anti-TAP) and with TAT-1 antibody as a loading control (anti-tubulin) (b). As a size marker the Spectra™ Multicolor Broad Range Protein Ladder (cat.#: 26634) was used.
